# Predictors of exclusive breastfeeding across three time points in Bangladesh: an examination of the 2007, 2011 and 2014 Demographic and Health Survey

**DOI:** 10.1093/inthealth/ihy015

**Published:** 2018-03-22

**Authors:** Sarah R Blackstone, Tina Sanghvi

**Affiliations:** 1 Department of Health Sciences, James Madison University, 235 Martin Luther King Jr. Way, MSC 4301, Harrisonburg, VA 22807, USA; 2 FHI 360, Washington, DC, USA

**Keywords:** Bangladesh, Exclusive breastfeeding

## Abstract

**Background:**

The objective of this study was to explore predictors of exclusive breastfeeding (EBF) in Bangladesh using data from 2007, 2011 and 2014, specifically focusing on potential reasons why rates of EBF changed over those time periods.

**Methods:**

Data on mother/infant pairs with infants <6 months of age were examined at the three time points using the Bangladesh Demographic and Health Survey. The EBF prevalence, changes in EBF since the previous survey and determinants of EBF at each time period were examined using t-tests, χ^2^ and multilevel logistic regression.

**Results:**

The prevalence of EBF was 42.5, 65 and 59.4% in 2007, 2011 and 2014, respectively. The age of the child was significantly associated with EBF across all time points. The largest changes in EBF occurred in the 3- to 5-month age group. Predictors of EBF in this specific age group were similar to overall predictors (e.g. age of the child and region). Participation of the mother in household decisions was a significant predictor in 2014.

**Conclusions:**

EBF prevalence in Bangladesh increased between 2007 and 2011 and then decreased between 2011 and 2014. The increase in 2011 may have been the result of widespread initiatives to promote EBF in that time frame. Due to the unexplained decrease in EBF between 2011 and 2014, there is still a need for interventions such as peer counselling, antenatal education and community awareness to promote EBF.

Exclusive breastfeeding (EBF), defined as administering only breast milk and no other liquids or fluids or foods,^1^ is estimated to have wide-ranging impacts on mortality, morbidity, cognition and women’s health.^2^ In fact, the scaling up of breastfeeding to a near universal level could prevent 823 000 annual deaths in children <5 y of age and 20 000 annual deaths from breast cancer.^2^ EBF reduces the risk of gastrointestinal diseases and acute respiratory infections, which are some of the leading causes of death among young children.^2,3^ Long-term benefits of EBF include better school performance, productivity, and intellectual and social development. High-income countries have shorter breastfeeding durations than low- and middle-income countries. However, even in low- and middle-income countries, only 37% of infants <6 months of age are exclusively breastfed.^2^ In Southeast Asia, rates of EBF from 0 to 6 months range from 15.1% to 17.0% in Vietnam and Thailand, respectively, to 73.5% in Cambodia,^4^ typically with smaller, lower-income countries having a higher prevalence of EBF.^5^ In Bangladesh, rates of EBF were estimated at 59.4% in 2014.^6^ While most infants in Southeast Asia do receive breast milk to some extent during the first 6 months, with rates ranging from 88.5% in Indonesia and 97.5% in Timor-Leste to 61% in Thailand,^4^ not all infants who receive breast milk are exclusively breastfed. Although rates of EBF in this region are increasing, there is still a large proportion of infants not meeting the World Health Organization (WHO) recommendations for breastfeeding.^4^

In an attempt to understand why the rates of EBF are suboptimal in Southeast Asia, several studies have investigated different barriers that could impact the willingness or ability of mothers to exclusively breastfeed their children. Cultural practices such as feeding children additional foods and liquids, including honey, sugar water or mustard oil, immediately after birth were identified as contributing to lower rates of EBF.^7^ Furthermore, many mothers are working due to financial constraints on their families. Unfavourable work environments that either prohibit breastfeeding or prohibit mothers from leaving work to breastfeed contribute to lower rates of EBF. Evidence indicates that women who are of lower socio-economic status and unemployed are more likely to exclusively breastfeed because they do not encounter workplace and employment restrictions.^8^ This also contributes to the fact that lower-income countries, where greater proportions of women do not enter the formal workforce, typically have higher rates of EBF.^4^ In a comparative study of working and non-working mothers in Bangladesh, Hassan et al.^9^ found that 78% of working mothers continued EBF up to 3 months and 21% at 6 months. Among non-working mothers, 66% continued EBF at 3 months and 45% at 6 months, suggesting that during the first few months of maternity leave, working mothers are able to breastfeed but have more difficulty doing so after returning to work 3–4 months post-partum. Other studies throughout Southeast Asia have supported this as well. In Vietnam, Indonesia, Cambodia, the Philippines and Timor-Leste, higher levels of education, as well as maternal age, were predictors of not exclusively breastfeeding.^10^

Inadequate knowledge of appropriate breastfeeding practices and poor understanding of what EBF entails (e.g. no extra water) have also been cited as reasons for poor rates of EBF.^9,11^ A study in Nepal evaluating a breastfeeding promotion programme showed that only 35% of women received 100% of the breastfeeding promotional material (e.g. the importance of breastfeeding on demand and not to provide pacifiers or teats). Receiving more types of breastfeeding promotional material and information and from multiple different channels was associated with a decreased risk of early cessation of breastfeeding.^12^ Additionally, knowledge of the benefits of breastfeeding and advice from home health professionals significantly influence women’s decisions to breastfeed and should be taken into consideration to better tailor breastfeeding promotion.^13^ For instance, in northern India, Mahmood et al.^14^ found that 47% of the women interviewed were not aware of the lifesaving benefits of EBF and, as a result, began introducing complementary foods before 6 months of age. Inadequate post-natal care has shown associations with non-EBF as well.^10^ At the health policy level, a lack of comprehensive breastfeeding promotion programmes and inadequate maternity leave also contribute to suboptimal rates of EBF.^4^ In Vietnam and Bangladesh comprehensive breastfeeding promotion strategies led to rapid large-scale increases in breastfeeding.^15^

There have been many initiatives to improve EBF and other infant and young child feeding practices in Bangladesh, including the Baby Friendly Hospital Initiative,^16^ the National Strategy for Infant and Young Child Feeding in Bangladesh^17^ and the World Breastfeeding Trends Initiative (WBTi).^18^ More recently, Alive & Thrive improved maternal nutrition and infant and young child feeding practices by increasing coverage of maternal nutrition interventions, exclusive breastfeeding and complementary feeding practices. Alive & Thrive began its implementation in 2009 and continued through 2014. Results in Bangladesh indicated that EBF increased from 47% in 2010 to >80% in 2014 in the areas where the programme was implemented.^19^ Although these results are promising, it is necessary to look at the national level to see if, and how, trends in EBF have changed overtime. The Demographic and Health Survey (DHS) in Bangladesh provides a rich source of data for understanding the shifts in EBF since the implementation of Alive & Thrive and the implementation of other programmes. Thus the purpose of this study was to use the Bangladesh DHS to estimate the trends in EBF and the individual-, household- and community-level determinants that may have contributed in the last three waves of the DHS in 2007, 2011 and 2014. Given the health benefits associated with EBF and the gap in the literature regarding time trends of EBF and associated factors in Bangladesh, this study can provide valuable information to inform health promotion programming and health policy related to improving breastfeeding practices and child health.

## Methods

The current cross-sectional data came from three waves of the Bangladesh DHS in 2007, 2011 and 2014. The Bangladesh DHS uses a sampling frame from the list of enumeration areas (EAs) designated by the 2001 population census for the 2007 survey and the 2011 population and housing census of the People’s Republic of Bangladesh, provided by the Bangladesh Bureau of Statistics (BBS) in 2011 and 2014. EAs are the primary sampling unit and contain approximately 120 households. The 2011 and 2014 DHS used a two-stage stratified sample of public and private households. Details of the sampling strategy are published elsewhere. Briefly, trained field workers conducted face-to-face interviews with adult members of 11 485 households in 2007, 17 511 households in 2011 and 17 300 households in 2014. Women 12–49 y of age were eligible to answer questionnaires on reproductive history, infant and young child feeding practices, antenatal care and fertility preferences, among others. Background information was also collected for each participant (e.g. age, education, place of residence and wealth).^6,20^

## Exclusive breastfeeding

A variable measuring EBF was computed in two stages. First, mothers reported whether their infant was breastfed (yes/no). Second, mothers reported whether they gave their child food from the following categories: other liquids (e.g. juice, water, formula), grains, legumes, dairy, meat, fruits, vegetables and sweets. A binary variable was created that delineated whether a mother gave any of the aforementioned complementary foods or not. The EBF variable was subsequently computed by combining mothers’ reported breastfeeding and complementary feeding; mothers who exclusively breastfed were coded as 1, while mothers who did not breastfeed and mothers who breastfed but also gave complementary foods were coded as 0.

### Predictors

#### Household factors

The family wealth index and region of residence were examined in relation to EBF. The family wealth index is a scale variable computed by the DHS programme based on household location, income and amenities. The index is divided into five quintiles. For this study we combined the two lowest and the two highest wealth quintiles due to limited observations, for a total of three categories: lowest wealth index, middle wealth index and highest wealth index. The region of residence was examined by dummy coding each of the seven political districts of Bangladesh. For each year, the reference category was the region with the highest prevalence of EBF.

#### Antenatal care and delivery

Two indicators were used as proxies of antenatal and delivery practices. Women were asked how many antenatal visits they attended during their pregnancy, which was treated as a continuous predictor variable. Women were also asked where delivery took place for their last birth (e.g. home, hospital, birthing centre, etc.). This was recoded as a binary variable: delivered in a health facility (including hospitals, birthing centres, health clinics, etc.) or delivered at home. Women reported the type of health care provider they saw for both antenatal care and delivery. These two separate variables were recoded as binary variables: antenatal care and delivery, respectively, given by a skilled health care provider (physician, nurse or midwife) or not.

#### Women’s status

Education, exposure to information, employment and decision making were assessed as predictors of EBF. Education was measured by the highest level of education the women had received (no education, primary, secondary, tertiary or higher). Because of limited observations in the tertiary category, we combined secondary and tertiary education for a total of three education categories. Exposure to newspapers, television and radio were proxies for exposure to information. Women reported the frequency of being exposed to newspapers, television and radio. These were recorded indicating exposure once a week or more or less than once a week for each variable. A binary variable measuring employment was developed (0=not employed in the last 12 months; 1=employed in the last 12 months). Due to the limited number of respondents who worked in the last 12 months, any type of employment (e.g. part time, seasonal or full time) was considered employed for the purposes of the variable. Finally, a decision-making scale was created based on women’s involvement in decisions regarding health care, household purchases, visiting family and friends and who spends the respondent’s earnings. This scale ranged from 0 to 4 based on the number of decisions in which respondents were involved.

### Statistical analysis

#### Descriptive statistics

Prevalence of EBF was compared for each year overall, as well as for each age group (1 month, 2 months, etc.). t-tests were used to compare EBF between 2007 and 2011, 2007 and 2014, and 2011 and 2014, as well as EBF in each age group over the three time points.

#### Bivariate analyses

Bivariate analyses were conducted to examine relationships between the aforementioned variables and EBF in 2007, 2011 and 2014. Variables that were significant in bivariate analyses were included in the final logistic regression models. After significant bivariate associations were identified, the researchers compared the variables between 2007, 2011 and 2014 to examine whether the distribution of these variables differed significantly in the three samples. The final predictors chosen based on bivariate results were education, decision making, place of delivery and region. In comparing the final predictors across the three time points, we found that education in 2011 and 2014 was significantly higher than in 2007. Delivery in a health facility was significantly higher in 2011 and 2014 than in 2007 and higher in 2014 than in 2011. Decision making was not significantly different between the three time points, nor was the proportion of the sample living in each region of Bangladesh. Bivariate results and cross-tabulations are available in supplemental files.

#### Multivariable analysis

Data were weighted using the svy set command in STATA (StataCorp, College Station, TX, USA) to estimate the prevalence and confidence intervals accounting for cluster sampling design. Multilevel binary logistic regression accounting for the region of residence was used to determine factors associated with EBF in 2007, 2011 and 2014. Multilevel models allow for control of random effects between different clusters of participants, which is why it was chosen for this analysis. Additionally, the age and gender of the child were controlled as confounders. We individually examined each model and computed to assess the adjusted risk of independent variables. Statistical significance was determined as p-values <0.05. The researchers examined significant factors in 2007, 2011 and 2014 to understand which variables may have contributed to a change in EBF between the three time points. All analyses were conducted with STATA 14.

## Results

### Descriptive statistics

**Figure 1.**
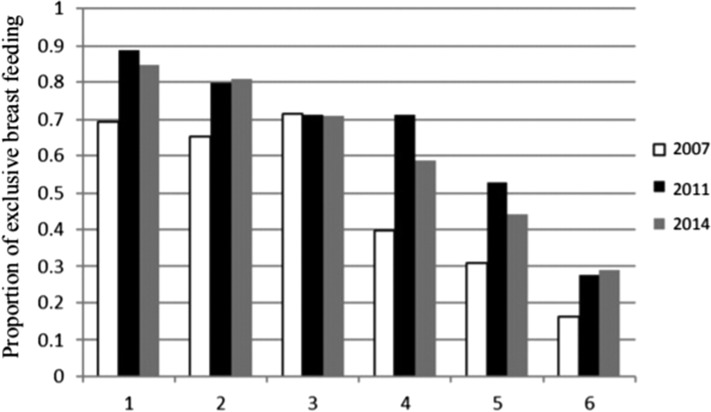
Proportion of EBF by age group and year.

Infants 0–5 months were included in the analysis (n=515 in 2007, n=800 in 2011, n=638 in 2014). The prevalence of EBF was 42.5, 65 and 59.4% in 2007, 2011 and 2014, respectively. EBF in 2007 was significant less than EBF in 2011 (t=8.1, p<0.001) and 2014 (t=2.97, p<0.01). EBF in 2014 was significantly less than EBF in 2011 (t=5.49, p<0.01). See Figure 1 and Table 1 for the prevalence of EBF by infant age in months for each respective year. Significant increases were seen between 2007 and 2011 among infants 0 (t=2.73, p<0.01), 1 (t=2.63, p<0.01) and 4 (t=2.26, p<0.05) months old and a significant decrease in EBF among 3-month-olds (t=3.2, p<0.01). Between 2011 and 2014 there was a significant increase in EBF among 3-month-olds (t=2.04, p<0.05). See Table 2 for sample demographics.

**Table 1.** EBF comparison by age groups over time

Age group	2007–2011	2007–2014	2011–2014
0	2.73**	1.83	−0.811
1	2.63**	2.55*	0.021
2	1.72	1.61	−0.049
3	4.78***	2.63**	−2.04*
4	3.21**	1.84	−1.34
5	2.26*	2.44*	0.215

*If negative, EBF decreased between the first time point and the second.

**Table 2.** Sample demographics

Variable	2007	2011	2014
EBF	219 (42.5)	530 (65)	379 (59.4)
Employed	85 (8.9)	44 (5.5)	109 (17)
Skilled delivery attendant	146 (28.4)	313 (74.8)	298 (66.5)
Skilled ANC attendant	71 (19.9)	367 (49.2)	394 (61.9)
Barisal resident	65 (12.6)	74 (9.3)	91 (14.3)
Chittagong resident	111 (21.6)	174 (21.8)	126 (19.75)
Dhaka resident	94 (18.2)	132 (16.5)	104 (16.3)
Khulna resident	54 (10.5)	112 (14.0)	71 (11.1)
Rajshahi resident	85 (16.5)	102 (12.8)	72 (11.3)
Rangpur resident	N/A	94 (11.8)	77 (12.1)
Sylhet resident	106 (20.6)	112 (14)	97 (15.2)
Rural residents	337 (65.4)	547 (68.4)	433 (67.8)
Health centre delivery	146 (28.4)	295 (36.9)	294 (46.2)
Male child	260 (50.5)	414 (51.8)	346 (54.2)
Women involved in ≥3 decisions	258 (50)	304 (38)	260 (40.8)
Number of ANC visits, mean (SD)	2.4 (2.6)	2.6 (2.6)	2.9 (2.6)
Number of children, mean (SD)	2.5 (1.7)	2.3 (1.5)	2.1 (1.4)

Values presented as n (%) unless stated otherwise.

ANC: antenatal care; N/A: not available; SD: standard deviation.

In 2007 among children 0–5 months of age, significant predictors of EBF were the age of the child (β=−0.498, p<0.001); older children were less likely to be exclusively breastfed. Delivery in a health facility was moderately associated with EBF (β=0.554, p=0.05). In 2011, the age of the child was significantly associated with EBF, with older children having lower odds of EBF (β=−0.568, p<0.001). Finally, in 2014, involvement in decision making was positively associated with EBF (β=0.198, p<0.01). See Table 3.

**Table 3.** Predictors of EBF at 0–5 months

	2007		2011		2014	
Predictor	β	95% CI	β	95% CI	β	95% CI
Decision making	0.019	−132 to 0.171	0.025	−0.149 to 0.201	0.198*	0.019 to 0.377
Place of delivery						
Home (ref)						
Health facility	0.554 (p=0.05)	−0.042 to 1.39	0.253	−0.250 to 0.757	−0.138	−0.943 to 0.667
Delivery attendant						
Not skilled (ref)						
Skilled	0.364	−0.191 to 0.920	0.006	−0.056 to 0.068	0.229	−0.581 to 1.04
ANC attendant						
Not skilled (ref)						
Skilled	0.122	−0.477 to 0.721	−0.285	−0.784 to 0.213	0.166	−0.356 to 0.688
Number of ANC visits	−0.057	−0.166 to 0.052	−0.009	−0.098 to 0.078	0.019	−0.074 to 0.114
Employment						
Not employed (ref)						
Employed	−0.291	−0.963 to 0.380	0.470	−0.367 to 1.31	0.145	−0.438 to 0.729
Parity	0.029	−136 to 0.196	−0.144	−0.345 to 0.523	0.040	−0.155 to 0.235
Sex of child						
Male (ref)						
Female	0.252	−0.218 to 0.723	0.088	−0.345 to 0.523	0.175	−0.269 to 0.621
Age of child	−0.498***	−0.648 to 347	−0.430***	−0.573 to −0.289	−0.637***	−0.791 to −0.484

ANC: antenatal care; ref: reference.

*p<0.05; **p<0.01; ***p<0.001.

Because the greatest changes in EBF over the three time points were seen in the 3- to 5-month age group, we also examined predictors of EBF specifically among infants ages 3–5 months. In 2007, 2011 and 2014, the age of the child was associated with EBF; older children were less likely to be exclusively breastfed. In 2007, delivery in a health facility was positively associated with EBF (β=0.1.89, p<0.01). In 2014, decision making was positively associated with EBF (β=0.272, p<0.01). See Table 4.

**Table 4.** Predictors of EBF in infants 3–5 months of age

	2007		2011		2014	
	β	95% CI	β	95% CI	β	95% CI
Decision making	0.077	−0.105 to 259	0.012	−0.237 to 0.262	0.272**	0.030 to 0.514
Place of delivery						
Home (ref)						
Health facility	1.89**	0.567 to 3.21	0.672	−0.253 to 1.59	−0.810	−2.01 to 0.386
Delivery attendant						
Not skilled (ref)						
Skilled	−0.968	−2.22 to 0.284	−0.193	−1.22 to 0.835	0.620	−0.568 to 1.81
ANC attendant						
Not skilled (ref)						
Skilled	−0.149	−1.46 to 1.17	0.012	−0.697 to 0.721	0.070	−0.650 to 0.791
Number of ANC visits	−0.051	−0.259 to 0.156	−0.019	−0.146 to 0.107	0.110	−0.017 to 0.237
Employment						
Unemployed (ref)						
Employed	−0.626	−1.42 to 0.173	0.263	−0.815 to 1.34	0.414	−0.327 to 1.15
Parity	−0.047	−0.216 to 0.774	−0.012	−0.011 to 0.283	−0.016	−0.252 to 0.231
Sex of child						
Male (ref)						
Female	0.271	−0.292 to 0.835	0.605	−0.030 to 1.24	0.008	−0.577 to 0.593
Age of child	−0.581**	−0.922 to −0.242	−1.15***	−1.56 to 726	−0.676***	−1.04 to −0.317

ANC: antenatal care; ref: reference.

*p<0.05; **p<0.01; ***p<0.001.

## Discussion

Early childhood nutrition is essential for the immediate and long-term health of children, and EBF has proven benefits in the first year of life.^2^ EBF needs to be practised for 6 months to obtain the benefits of lowering childhood death rates and preventing chronic and infectious diseases.^21^ Because of the benefits of EBF, the WHO has recommended that all children be exclusively breastfed for the first 6 months of life and then continue to be breastfed until 23 months. In this study, the prevalence of EBF in infants 0–5 months of age in 2007, 2011 and 2014 was 42.5, 65 and 59.4%, respectively, which is significantly lower than the WHO/United Nations Children’s Fund recommended level of universal EBF.^22–24^ Additionally, the national levels of EBF were much lower in 2014 than the prevalence of EBF in areas in which Alive & Thrive was implemented.^19^ Understanding the factors associated with declines in EBF in the first 6 months can provide useful information as programmes such as Alive & Thrive continue to expand, identifying specific factors to focus on or subgroups in the population most at risk for suboptimal EBF.

In 2007 there was a moderately significant association between the place of delivery and EBF among infants 3–5 months of age. This complements evidence of the association between receiving breastfeeding promotional materials and EBF.^25–27^ In Bangladesh, as part of the Baby Friendly Hospital Initiative (BFHI), women who delivered in health care facilities received breastfeeding education and promotional materials and were more likely to engage in EBF. However, we did not find this in the 2011 Bangladesh DHS, perhaps because of less exposure to education and promotional materials. In 2014, among infants 3–5 months of age, delivery in a health facility was negatively associated with EBF, although not statistically significant. In Nepal, where BFHI did not achieve high coverage, this trend was seen as well; women who delivered at home were more likely to exclusively breastfeed than mothers delivering in a health facility.^28^ Other reasons for this include that women who are poorer are more likely to deliver at home and may not have funds for infant formula or other supplements, and thus are more likely to exclusively breastfeed.

Participation in decision making was associated with EBF in the 2014 Bangladesh DHS overall and among infants 3–5 months of age; decision making has been noted as one of the important aspects of women’s empowerment,^29^ defined as ‘the expansion of people’s ability to make strategic life choices in a context where this ability was previously denied to them’,^29^ and women’s ability to influence decision making in social, economic, familial and legal dimensions.^30^ Previous studies have shown that different dimensions of women’s empowerment, such as decision making, land ownership, education and employment, are associated with better child feeding practices and child nutrition, as well as expenditures on children’s needs and education.^31–33^ Thus, significant predictors of EBF shifted from delivery-related factors in 2007 to empowerment-related aspects in 2011 and 2014. Women’s empowerment is generally linked with improved health outcomes worldwide, including contraceptive use,^34^ antenatal care, use of skilled delivery attendants and post-natal care.^35–37^ The hypothesized reason for this is that women who are more empowered typically have better education opportunities, more access to health information and a greater ability to interpret and act on health information.^38^ With regard to EBF, empowered women are hypothesized to have better access to health care, better understanding of the benefits of EBF and more autonomy in making health care decisions. Decision making, an important aspect of women’s empowerment,^37^ was significant in 2014, suggesting that similar mechanisms may be driving the relationship between decision making and EBF in Bangladesh.

Indeed, women’s empowerment has more recently been noted in other studies as an important factor for promoting EBF. For instance, Kupratakul et al.^39^ investigated the impact of knowledge-sharing practices with empowerment strategies on breastfeeding for 6 months post-partum. Women in the intervention group received education and empowerment strategies to increase their autonomy, while women in the control group received only education on breastfeeding techniques. The authors found that women receiving education on empowerment strategies were significantly more likely to exclusively breastfeed at all follow-up points, including 5 and 6 months. Because of the significance of aspects of women’s empowerment in this study and others for EBF, future programmes aimed at increasing the prevalence and duration of EBF may begin to incorporate strategies to increase women’s empowerment.

Concerning the decrease in EBF between 2011 and 2014, while we cannot say definitively the reason for this decline, it is possible that this had to do with the increased employment rate in 2014 compared with 2011. Although employment was not significant in the final regression models, it was significantly different at the three time points. Employment was significantly greater in 2007 than 2011 and significantly greater in 2014 than 2011. Thus one reason for the changes in EBF trends between these time points could be employment in new mothers. Typically, mothers who are employed are less likely to exclusively breastfeed because they face workplace restrictions that limit the amount of time they are able to breastfeed.^25,40^ Because maternal employment was higher in 2007 (22%) and 2014 (24%) than in 2011 (10%),^6,20^ it is possible that this contributed to the increase in EBF between 2007 and 2011 and the decrease between 2011 and 2014. However, employment was not a significant factor in our model and therefore this must be interpreted with caution.

This is contradictory to studies demonstrating that employment is significantly linked with EBF.^4,24^ One of the reasons for this could be that the sample of employed women was relatively small and thus the analysis may not have had sufficient statistical power to yield significant results. Regardless, employment is associated with other aspects of women’s empowerment, such as education and decision making, and increases in employment opportunities actually have been shown to have a positive effect on women’s decision making in the household.^41^ Thus employment in addition to several other constructs related to women’s empowerment may be contributing to changes in EBF over time. If maternal employment indeed is in part responsible for the decrease in EBF between 2011 and 2014, working with employers to provide more opportunities for working mothers to breastfeed may be critical moving forward. It is important to note that these conclusions about maternal employment are beyond the scope of this study and something that other studies can consider. In investigating this, it is necessary to do studies with a more even distribution of employment in the sample to enable adequate statistical power.

There are several limitations to this study. First, the cross-sectional nature of the study prevents us from making any causal inferences. Additionally, all data collected were based on interviews and it is possible that recall bias and/or social desirability bias influenced responses. Another limitation is the use of 24-h recall information, which tends to overestimate the prevalence of EBF.^42^ Despite these limitations, this study did utilize data from a nationally representative source, promoting generalizability of the results. Additionally, we examined changes in determinants of EBF between 2007, 2011 and 2014, a time period in which EBF increased significantly and sharply decreased. Furthermore, we specifically investigated determinants in the 3- to 5-month age group, which showed the greatest changes in EBF over time.

These results have implications for intervention development, by highlighting key determinants that could be relevant for increasing EBF among children 0–5 months of age and can inform future recommendations. Future programmes should focus on promoting women’s empowerment to increase decision making and autonomy, as well as possibly targeting places of employment to offer ways for women to breastfeed after they have returned to work. Incorporating policy changes to encourage breastfeeding-friendly workplaces could impact this. There are several studies that indicate that a mother’s work environment has a strong impact on her decision to continue breastfeeding.^43,44^ Work-based lactation support programmes enhance a woman’s ability to adhere to EBF^45–48^ and is perceived favourably by women.^49^ Additionally, interventions utilizing empowerment strategies and focusing on ante- and post-natal knowledge-sharing practices and empowerment strategies have been shown to increase EBF in Southeast Asia,^39^ thus incorporating these strategies may be helpful for increasing EBF in Bangladesh.

## Supplementary data

Supplementary data are available at International Health Online (http://inthealth.oxfordjournals.org).

## Supplementary Material

Supplementary Data
