# Supplementary material for: Predictors of exclusive breastfeeding across three time points in Bangladesh: an examination of the 2007, 2011 and 2014 Demographic and Health Survey
Source: Int Health. 2018 Mar 22;10(3):149–56. doi: 10.1093/inthealth/ihy015 (PMC6537939; doi:10.1093/inthealth/ihy015)
Supplement: Supplementary Data [file ihy015supplementalfile.docx]

| **Bivariate analyses with EBF** | | | |
| --- | --- | --- | --- |
| *Variable* | *2007 (χ^2^)* | *2011 (χ^2^)* | *2014 (χ^2^)* |
| Colostrum | .064 | N/A | N/A |
| Skilled delivery attendant | 1.37 | 2.71 | .311 |
| Skilled antenatal provider | .445 | .025 | .285 |
| Decision making | 2.85 | .182 | 7.98* |
| Employment | 2.94* | .038 | .828 |
| Place of delivery | 1.36 | .269 | .363 |
| Antenatal | 9.64 | .523 | 9.35 |
| Urban/rural | .479 | .061 | 1.37 |

*p < .1

| **Given colostrum** | **EBF No** | **EBF yes** |
| --- | --- | --- |
| No | 22 | 15 |
| Yes | 274 | 204 |
| Total | 296 | 219 |

| **Skilled delivery attendant** | **EBF No** | **EBF Yes** |
| --- | --- | --- |
| **2007** | | |
| No | 218 | 78 |
| Yes | 151 | 68 |
| *Total* | 369 | 146 |
| **2011** | | |
| No | 37 | 104 |
| Yes | 66 | 209 |
| *Total* | 103 | 313 |
| **2014** | | |
| No | 63 | 117 |
| Yes | 87 | 181 |
| *Total* | 150 | 298 |

| **Skilled antenatal provider** | **EBF No** | **EBF Yes** |
| --- | --- | --- |
| **2007** | | |
| No | 161 | 37 |
| Yes | 124 | 34 |
| *Total* | 285 | 71 |
| **2011** | | |
| No | 147 | 128 |
| Yes | 281 | 239 |
| *Total* | 428 | 367 |
| **2014** | | |
| No | 101 | 156 |
| Yes | 141 | 238 |
| *Total* | 242 | 394 |

| **Decision making (# of decisions involved in)** | **EBF No** | **EBF Yes** |
| --- | --- | --- |
| **2007** | | |
| 0 | 62 | 44 |
| 1 | 42 | 35 |
| 2 | 44 | 30 |
| 3 | 148 | 110 |
| *Total* | 296 | 219 |
| **2011** |  |  |
| 0 | 92 | 168 |
| 1 | 43 | 89 |
| 2 | 41 | 72 |
| 3 | 194 | 200 |
| *Total* | 280 | 520 |
| **2014** | | |
| 0 | 93 | 110 |
| 1 | 44 | 50 |
| 2 | 29 | 51 |
| 3 | 93 | 167 |
| *Total* | 259 | 379 |

| **Employment** | **EBF No** | **EBF Yes** |
| --- | --- | --- |
| **2007** | | |
| No | 240 | 56 |
| Yes | 190 | 29 |
| *Total* | 430 | 85 |
| **2011** | | |
| No | 264 | 16 |
| Yes | 492 | 28 |
| *Total* | 756 | 44 |
| **2014** | | |
| No | 219 | 40 |
| Yes | 310 | 69 |
| *Total* | 529 | 109 |

| **Place of delivery** | **EBF No** | **EBF Yes** |
| --- | --- | --- |
| **2007** | | |
| Not health facility | 218 | 78 |
| Health facility | 151 | 68 |
| *Total* |  |  |
| **2011** | | |
| Not health facility | 180 | 100 |
| Health facility | 324 | 195 |
| *Total* | 504 | 295 |
| **2014** | | |
| Not health facility | 143 | 116 |
| Health facility | 199 | 178 |
| *Total* | 342 | 178 |

| **Number of antenatal visits** | **EBF No** | **EBF Yes** |
| --- | --- | --- |
| **2007** | | |
| Less than 4 | 220 | 165 |
| More than 4 | 73 | 54 |
| *Total* | 293 | 219 |
| **2011** | | |
| Less than 4 | 203 | 356 |
| More than 4 | 73 | 170 |
| *Total* | 276 | 526 |
| **2014** | | |
| Less than 4 | 168 | 87 |
| More than 4 | 255 | 124 |
| *Total* | 423 | 211 |

| **Urban/rural** | **EBF No** | **EBF Yes** |
| --- | --- | --- |
| **2007** | | |
| Urban | 106 | 72 |
| Rural | 190 | 147 |
| *Total* | 296 | 219 |
| **2011** | | |
| Urban | 87 | 166 |
| Rural | 193 | 354 |
| *Total* | 280 | 520 |
| **2014** | | |
| Urban | 90 | 115 |
| Rural | 169 | 264 |
| *Total* | 259 | 379 |
